# Does the Majority Always Know Best? Young Children's Flexible Trust in Majority Opinion

**DOI:** 10.1371/journal.pone.0104585

**Published:** 2014-08-12

**Authors:** Shiri Einav

**Affiliations:** School of Psychology, University of Nottingham, Nottingham, United Kingdom; Durham University, United Kingdom

## Abstract

Copying the majority is generally an adaptive social learning strategy but the majority does not always know best. Previous work has demonstrated young children's selective uptake of information from a consensus over a lone dissenter. The current study examined children's flexibility in following the majority: do they overextend their reliance on this heuristic to situations where the dissenting individual has privileged knowledge and should be trusted instead? Four- to six- year-olds (N = 103) heard conflicting claims about the identity of hidden drawings from a majority and a dissenter in two between-subject conditions: in one, the dissenter had privileged knowledge over the majority (he drew the pictures); in the other he did not (they were drawn by an absent third party). Overall, children were less likely to trust the majority in the Privileged Dissenter condition. Moreover, 5- and 6- year-olds made majority-based inferences when the dissenter had no privileged knowledge but systematically endorsed the dissenter when he drew the pictures. The current findings suggest that by 5 years, children are able to make an epistemic-based judgment to decide whether or not to follow the majority rather than automatically following the most common view.

## Introduction

Think back to the last time you consulted customer reviews online to decide whether or not to purchase the latest bestseller, or that time you forgot your conference program and were not sure where to go for the Keynote Address. Chances are that in both cases you followed the majority. Although the reliability of others' testimony is variable, when a number of individuals converge on a particular view we feel more confident that it is correct and can be trusted ([1], see [2] for review). It is the shared perspective among multiple people that strengthens the credibility of the testimony. At the same time, we tend to place less weight on the dissenting opinion or actions of lone individuals (e.g., the one ‘poor’ compared to forty ‘excellent’ reviews on that novel, or the one person who heads toward the stairs while everyone else makes their way down the corridor at the conference). This is an adaptive heuristic as, in general, a view that is endorsed by several people is more reliable than the potentially idiosyncratic or false belief of one individual (see [3]–[5]). However, the majority does not always know best. There may be circumstances where a dissenting individual has privileged knowledge over the majority due to having expertise (e.g., a literary critic) or access to particular information that others are not privy to (e.g., a conference delegate who has heard that the Keynote Address has been moved to a different location due to a technical fault with the equipment). In such cases, it would be maladaptive to discount the dissenter in favor of the consensual view.

In line with mounting evidence of children's selective trust in testimony (e.g., [6], see [7] for review), recent findings suggest that sensitivity to social consensus constrains children's learning from a young age. Corriveau et al. [8] presented children with a language task where they were faced with conflicting information about the object referents of novel labels. Preschoolers selectively endorsed the majority view over the view of a lone dissenter (see also [9]). In the case of conventional knowledge such as language, where the maintenance of word meanings depends on shared agreement [10], following the majority is clearly the sensible thing to do. However, we do not currently know whether children overextend their reliance on this cue to situations where the dissenting individual has privileged knowledge over the majority and should therefore be trusted instead. Under such circumstances, they may continue to endorse the majority for a number of reasons: a preference to conform to the group; the greater salience of the more common view; a failure to recognize the dissenter's privileged knowledge; or a misguided belief that the majority are always correct.

Previous literature suggests that children can be flexible when applying certain criteria for selective trust (see [11] for review). For example, children generally prefer to learn from adults over other children; however, this preference is reversed when the adult informant is shown to be less accurate than the child, or when the knowledge domain pertains to child-relevant subject matter such as toys [12], [13]. Similarly, from the age of 4 years, children cease trusting a familiar over an unfamiliar speaker, as well as a speaker with a native-accent over one with a foreign accent (both default biases), if the familiar or native-accent speaker proves to be less accurate than the unfamiliar or foreign-accent speaker, respectively [14], [15]. Finally, 4-year-olds, who typically mistrust an informant who has been incorrect in the past, will learn from a previously inaccurate informant whose errors arose legitimately from inadequate information access [16].

The present study was designed to investigate children's flexibility in using consensus information. Specifically, we examined whether the heuristic to follow the majority would be overridden by an epistemic-based judgment that takes into account the underlying knowledge of informants. To find out, the majority was pitted against a lone dissenter in two between-subject conditions: in one the dissenter had privileged knowledge over the majority; in the other he did not. Would children rigidly side with the majority in both conditions, or would they refrain from doing so when the dissenter was in the best position to know the truth? We focused on 4- to 6- year-olds to capture the age range in which children have been shown to follow the majority, and to apply other trust heuristics flexibly according to context.

Participants were presented with conflicting testimony from four informants (three consensual claims vs. one dissenting claim) on the basis of which they had to guess the identity of hidden drawings. In the condition where the dissenter had privileged knowledge (PD), he drew the pictures whereas in the non-privileged dissenter (NPD) condition, the pictures had been drawn by an absent third party. Drawing provided a suitable context for our task for two reasons. The majority could be assumed to have relevant knowledge on the question at stake. They had seen the picture and could therefore be expected to know its identity. Nevertheless, the artist, by virtue of having created the picture, was in a privileged position to know what it depicted, i.e., his intention defined it. A circle on a page may look like a ball to several people but if the individual who drew it intended it to be an orange then the convention is to accept it as an orange. Knowledge about the importance of artist intent is acquired early. Preschoolers reason about the creator's intention when attributing labels to drawings [17]. Similarly, they are more likely to accept an unexpected label for an artifact when that label was provided by the artifact's creator than by someone who merely discovered it [18]. One could therefore assume that children in the PD condition would be aware of the artist's privileged knowledge compared to that of the other informants. Of interest was whether they would overcome the tendency to follow the majority and instead trust the claims of the dissenter when asked to infer what he drew.

In addition to the forced-choice response, participants were asked to justify their decision on every trial. This afforded an examination of whether they would explicitly verbalize their a) reliance on the majority and b) awareness of the dissenter's privileged knowledge in the PD condition. Although previous studies have demonstrated the early influence of the majority on children's trust, we do not currently know whether children can explicitly reflect on their use of this cue.

## Method

### Ethics statement

This research was approved by the University Research Ethics Committee at Oxford Brookes University, and was conducted in accordance with British Psychological Society ethical guidelines. Participants' parents provided written informed consent.

### Participants

One hundred and three children participated in the study. There were thirty-two 4-year-olds (*M* = 4;6, range 3;11 to 4;11; 14 boys and 18 girls), forty 5-year-olds (*M* = 5;5, range 5;0 to 5;11; 23 boys and 17 girls) and thirty-one 6-year-olds (*M* = 6;6, range  = 6;0 to 6;10; 18 boys and 13 girls). All children were native English speakers, recruited from four schools in predominantly White middle-class neighborhoods.

### Materials

Four child-like boy hand puppets with moveable mouths and hands acted as the informants. Puppets, presented live, were used in order to make the task as interactive and engaging as possible for participants. (See e.g., [19], [20] for previous studies that have demonstrated children's selective trust while using puppets as the informants.) There were eight picture cards that matched up in pairs according to shape (orange/ball, snake/rope, crescent moon/banana, bat/pencil), a wall used as an occluder, a pen and small sheets of plain paper, and an envelope addressed to the experimenter (E) containing plain paper for the NPD condition.

### Design and Procedure

Participants were tested individually in a quiet area near their classroom. In each age group, participants were randomly assigned to either the *privileged dissenter* (PD) or *non-privileged dissenter* (NPD) condition. Testing began with a *sorting warmup* to introduce children to the stimuli and set the context for the ambiguity of the drawings and conflicting responses given by the informants in the trust task. Eight picture cards were placed in a random order on the table and children were asked to sort them into pairs according to shape. All children were able to complete this successfully. E pointed to each of the pairs in turn, confirmed that it was correct and asked children why the pictures went together. All children responded correctly either verbally e.g., “because they are both round” or by producing a gesture indicating their similar shape, e.g., tracing the squiggly contour of the snake and rope.

The *trust task* followed. Children were introduced to four puppet informants who would play a game with them. E put all the picture pairs to one side except for the orange/ball pair and asked the child, “Can you tell me what these things are?” All children were able to label the pictures correctly. The procedure then differed according to condition:

In the PD condition, E held up one of the puppets (designated as the drawing dissenter throughout the trials) and said, “This puppet is going to draw one of these pictures, either a ball or an orange, but he is going to do it behind this wall [puts up occluder] so you won't be able to see which one he draws. But after he finishes, all the puppets will have a look at the picture and we'll ask them what he drew, and then you can guess at the end.” E cleared away the orange and ball pictures, handed the puppet a pen and placed a piece of paper behind the occluder. The puppet then ‘drew’ the picture out of the child's sight. When it was finished children watched as each of the puppets in turn was shown behind the barrier and asked, “What did he/you draw?” Note that each puppet answered separately while the other puppets were placed behind E's back “so they won't be able to hear anything”. The drawing puppet gave one response, “an orange” while the other three puppets gave the same alternative response, “a ball”. The order in which the puppets responded (majority followed by dissenter or vice versa) alternated across the four trials, with half of the children in each age group and condition viewing a MD–DM–MD–DM sequence and the other a DM–MD–DM–MD sequence (where M =  majority, and D =  dissenter). The specific label endorsed by the majority and the drawing dissenter, respectively, and the identity of the drawing dissenter were also counterbalanced across participants. Children were given a summary of the informants' claims: “So he said he drew [an orange] and they said he drew [a ball]” in the same order as the speakers had responded. Children were then asked the *Test question*: “Now it's your turn to decide, what do you think he drew, [an orange] or [a ball]?” Participants were also asked to justify their decision, “Why do you think that?” No feedback was given except for neutral encouragement. The above procedure was repeated for three further trials using the remaining picture pairs.

In the NPD condition, E also kept the orange/ball picture pair in front of the participant and said, “My brother Larry really likes to draw pictures. He drew some pictures for me and sent them over in the mail because he lives far away. I asked Larry to draw a picture of either a ball or an orange. I wonder which one he drew.” E then held up one of the puppets (designated as the helping dissenter throughout the trials) and said to it, “Can you please get the envelope Larry sent me?” The puppet replied, “OK!” rummaged around in E's bag and brought out the envelope saying “Here it is!” E then explained, “Now in this game, I'm going to put this wall up [puts up occluder] and this puppet will put the picture that Larry drew behind the wall so you won't be able to see what it is. But then all the puppets will have a look at the picture and we'll ask them what he drew, and you can guess at the end.” The puppet pulled out the drawing from the envelope and put it down behind the occluder where it could not be seen by the child. In this way, the dissenting puppet's association with the pictures matched the PD condition, the only difference being that in the PD condition the puppet drew the pictures whereas in the NPD condition he only handled them. Children then watched as each of the puppets in turn was shown behind the barrier and asked “What did Larry draw?” The puppets' responses and counterbalancing procedures were identical to the PD condition. Children were asked the *Test question*, “So he said Larry drew [an orange] and they said Larry drew [a ball]. What do you think Larry drew, [an orange] or [a ball]?” In addition, participants were asked to justify their decision. The above procedure was repeated for three further trials using the remaining picture pairs.

### Coding

The main interest in looking at children's justifications was to examine the rates at which they would refer to the authority of the majority and/or the knowledge of the privileged dissenter. Responses were only rated as such if children referred to the frequency of the majority opinion (e.g., “More voted for it”; “Three of them said it”) or to the fact that the dissenter drew the picture (e.g., “He knows what he's drawn”; “He drew it, they don't really know”). Answers that simply referred to the identity of the speaker who said the same as the child without showing further insight were not included (e.g., “Because he said it”; “They said it.”) but were coded separately. Alternative explanations in both conditions fell into one of the following categories: description of the hidden drawing (e.g., “Because bananas are healthy”) or residual (any other response, including “Because they're lying”; “Because it is”; “Don't know” or no response). This coding scheme was used to individually code every trial for each participant. A second independent rater coded half of the responses (52 participants ×4 trials  = 208 justifications) and interrater agreement (agreements/agreements + disagreements) was 94%. Disagreements were resolved by discussion. Participants received an overall categorization that reflected his or her most frequent explanation type across the four trials. Participants who cited two or more explanation types with the same frequency were classified as ‘mixed’. However, if authority of the majority/drawer was one of these explanation types (e.g., two ‘majority/drawer’ justifications and two ‘they/he said it’ justifications), the majority/drawer classification was given to avoid underrating participants' awareness of these relevant criteria.

## Results

### Judgments

Participants received 1 point every time they endorsed the claim made by the majority for a maximum of 4 points. Preliminary analysis indicated no significant effects of gender, counterbalancing sequence or identity of dissenter puppet; therefore, the data were collapsed on these dimensions. Table 1 presents the mean number of times participants endorsed the majority view when guessing the identity of the pictures by age and condition. Table 2 shows performance on an individual basis, i.e., the number of participants who predominantly endorsed the majority, the dissenter, or were ambivalent across the 4 trials.

**Table 1:** Mean number of times participants endorsed the majority view (maximum score  = 4) by age and condition and comparisons against chance performance.

	Condition					
	Privileged dissenter			Non-privileged dissenter		
Age group	Mean (SD)	t	d	Mean (SD)	t	d
4-year-olds	1.06 (1.48)	−2.53*	−.64	2.31 (1.40)	.89	.22
5-year-olds	1.30 (1.49)	−2.10*	−.47	3.15 (1.14)**	4.52	1.01
6-year-olds	0.56 (1.15)	−4.99**	−1.25	3.47 (1.13)**	5.05	1.30

*p<.05;

**p<.001 when comparing with a chance score of 2.

**Table 2:** Number of participants who predominantly sided with the majority, the dissenter or were ambivalent in the privileged dissenter (PD) and non-privileged dissenter (NPD) conditions by age.

	PD			NPD		
	Dissenter	Ambivalent	Majority	Dissenter	Ambivalent	Majority
Age group						
4-year-olds	11	2	3	6	3	7
5-year-olds	12	4	4	2	2	16
6-year-olds	13	2	1	2	1	12

A 3 (age: 4 year-olds, 5-year-olds, 6 year-olds) ×2 (condition: PD, NPD) between-subjects analysis of variance (ANOVA) was conducted on participants' scores. The ANOVA confirmed a main effect of condition, *F*(1, 97)  = 59.20, *p*<.001, η^2^ = .38, with participants overall being less likely to side with the majority when the dissenting puppet had privileged knowledge (*M* = 1.00, *SD* = 1.40) than when it did not (*M* = 2.98, *SD* = 1.29). There was no main effect of age, *F*(2, 97)  = 1.50, *p* = .23, η^2^ = .03. The interaction between age and condition was significant, *F*(2, 97)  = 3.21, *p* = .045, η^2^ = .06. To interpret the interaction, the simple effect of condition was calculated for each age group. All three age groups were significantly less likely to side with the majority in the PD than the NPD condition: 4-year-olds, *F*(1, 97)  = 7.27, *p* = .008, η^2^ = .07; 5-year-olds, *F*(1, 97)  = 19.90, *p*<.001, η^2^ = .17; 6-year-olds, *F*(1, 97)  = 37.97, *p*<.001, η^2^ = .28. In addition, the simple effect of age was calculated separately for each condition. This effect was significant in the NPD condition, *F*(2, 97)  = 3.27, *p* = .042, η^2^ = .06, but not in the PD condition *F*(2, 97)  = 1.43, *p* = .24, η^2^ = .03. Pairwise comparisons with Bonferroni adjustment showed that 6-year-olds were more likely to endorse the majority in the NPD condition than 4-year-olds. The remaining comparisons were not significant.

Planned comparisons against a chance score of 2 (probability of success  = ½ for each trial multiplied by 4) are also shown on Table 1. These found that 5- and 6- year-olds made majority-based inferences when the dissenter had no privileged knowledge but systematically endorsed the dissenter when it drew the pictures (all *p*s<.05). In contrast, 4-year-olds systematically endorsed the drawing dissenter in the PD condition but did not perform differently to chance in the NPD condition.

Finally, performance did not differ systematically across trials in either condition. The percentage of children who endorsed the majority in the NPD condition was 71%, 78%, 73% and 76% on the first, second, third, and fourth trials respectively, Cochran's *Q*(3)  = 1.67, *p* = .64. The percentage of children who endorsed the majority in the PD condition was 21%, 21%, 27% and 31% on the first, second, third, and fourth trials respectively, Cochran's *Q*(3)  = 3.86, *p* = .28.

### Justifications

When asked to justify their decision in the NPD condition, there was a sharp rise in children's ability to verbalize their use of the majority heuristic between 5 and 6 years of age. Many of the 6-year-olds (80%) referred to the authority of the majority as their most frequent explanation across the 4 trials, whereas younger children were much less likely to respond in this way (4-year-olds  = 38%; 5-year-olds  = 40%). In the PD condition, reference to the authority of the privileged dissenter increased steadily with age: 6.3% 4-year-olds, 25% 5-year-olds, and 50% 6-year-olds provided this as their most frequent explanation across trials. Very few children (8% overall) cited the majority opinion in the PD condition. Alternative explanations in both conditions referred to the identity of the speaker who gave the same answer as the child without showing further insight; gave a description of the hidden drawing or failed to give a meaningful justification. The number of times children justified their choice with reference to the majority was positively correlated with the number of times they endorsed the majority's opinion, *r*(101)  = .70, *p*<.001.

## Discussion

Previous studies have shown that children recognize agreement among individuals and are more likely to copy information provided by the majority than a dissenter [8], [9], [21]. The current findings are the first to demonstrate that trust in the majority is flexible in children aged 5 and 6 years. As expected, they showed higher levels of trust in the majority over the dissenter about the identity of hidden pictures that were drawn by an absent third party and were therefore equally unfamiliar to all of the informants. By contrast, when the dissenter had privileged knowledge about the pictures by virtue of having drawn them, children selectively trusted his testimony even though it conflicted with the majority. These findings are consistent with previous findings showing that children can be flexible when applying criteria for trust by taking into account informants' past accuracy or their underlying knowledge (e.g., [12], [16]). As Wood et al. [11] note this flexibility may be important because it enables children to “continually source and copy the ‘best’ model” (p. 346).

Although 4-year-olds were less likely to endorse the majority in the PD compared to the NPD condition, they did not systematically favor the majority view in the NPD condition, suggesting that they did not generally accord the majority special authority. This result is surprising given preschoolers' preference for the majority in Corriveau et al. 's study [8]. However, it is consistent with recent data by Seston Schillaci and Keleman [22]. In their study, 3- and 4- year-olds did not reliably agree with the majority when judging the functions of novel objects. In trying to account for their contrasting findings with Corriveau et al., the authors proposed that domain differences in the content of information to be learnt may play a role. Specifically, they suggested that children may show greater susceptibility to social cues like consensus when it comes to learning about socially constructed conventions such as object labels than when learning about less arbitrary object functions that offer children “some independent, objective basis for judgment” (p. 11). In the current study, there was no objective basis for judgment as the drawings were kept hidden from sight, precisely to avoid the child simply basing their decision on what the picture looked more like to them (piloting found this to be a common strategy). Moreover, the task did involve making judgments about object labels as in Corriveau et al. Thus, Seston Schillaci and Keleman's explanation cannot account for the negative finding obtained here. An alternative explanation is that whereas in Corriveau et al. the informants' consensus was displayed via a simultaneous pointing cue, in the current study and in Seston Schillaci and Keleman's procedure, the testimony was conveyed verbally and sequentially. Perhaps when children first begin attending to agreement and disagreement among informants they require a salient visual depiction of individuals' views in order to form a representation of the agreement between them that is sufficiently strong to influence who they trust (see [22] for a related argument). Indeed, Haun et al. [21] found that children as young as 2 years were more likely to act on a novel apparatus in accordance with a behavior demonstrated by a majority of peers than the behavior of a dissenter. Therefore, it is also possible that by using action-based paradigms, researchers will uncover flexible trust in the majority at an earlier age than that demonstrated here. Nevertheless, the justifications data suggest that even though young children may be influenced implicitly by the majority, the development of children's explicit awareness of this heuristic (or at least their ability to verbalize it) is more protracted, becoming generally prevalent at 6 years of age.

In sum, children do not rigidly follow the most frequent opinion; they favor the minority view when it is likely to be more informed. The current findings suggest that by 5 years, children are able to make an epistemic-based judgment to decide whether or not to follow the majority. It is worth noting, however, that the respective weights of the majority and dissenter in any given situation may influence children's decisions. We only used one particular measure of majority consensus: the testimony of three puppets, instead of a group of peers. Under such conditions, children's conformist tendency is likely to have been driven primarily by ‘informational conformity’ (the motivation to obtain accurate information about reality) rather than ‘normative conformity’ (the motivation to maintain and develop group identity; for review see [2]). It is possible that in circumstances where normative conformity would be expected to play a greater role (i.e., inducing a strong desire to belong to the group), children would be less flexible in dismissing the majority view despite attributing greater knowledge to the dissenter. Finally, privileged knowledge is just one reason why an individual's claim may be more reliable than that of the majority. Future research should seek to establish whether children's trust in the majority would be similarly moderated in other situations where it would be wiser to listen to the lone voice in the crowd.
